# Identification of a novel HIV-1 third-generation circulating recombinant form (CRF126_0755) in Guangdong, China

**DOI:** 10.1007/s00705-024-06030-6

**Published:** 2024-04-08

**Authors:** Yun Lan, Ruolei Xin, Ruiying He, Feng Li, Xuemei Ling, Linghua Li, Fengyu Hu

**Affiliations:** 1 Institute of Infectious Diseases, Guangzhou Eighth People’s Hospital, Guangzhou Medical University, 8 Huaying Road, Baiyun District, Guangzhou, 510440 China; 2 Beijing Center for Disease Prevention and Control, Institute of AIDS/STD Prevention and Control, Beijing, 100013 China; 3 Guangzhou Medical Research Institute of Infectious Diseases, Infectious Disease Center, Guangzhou Eighth People’s Hospital, Guangzhou Medical University, 8 Huaying Road, Baiyun District, Guangzhou, 510440 China; 4 Guangdong Center for Diagnosis and Treatment of AIDS, Guangzhou, 510440 China

## Abstract

The genetic recombination patterns and genetic distribution of HIV-1 are valuable for elucidating the epidemic and genetic diversity of HIV. Numerous HIV-1 circulating recombinant forms (CRFs) have recently emerged and disseminated rapidly. In China, at least 32 CRFs have been reported to account for more than 80% of all HIV infections. However, CRFs derived from the CRF07_BC and CRF55_01B lineages have never been recorded. Here, a novel third-generation CRF involving HIV-1 was identified in four HIV-1-infected patients in Guangdong, China, who had no epidemiological association with each other. Phylogenetic and recombinant analyses confirmed that these strains shared highly similar recombination patterns, with the CRF07_BC backbone substituted by a CRF55_01B segment; therefore, these strains were classified as CRF126_0755. This is the first study of a CRF derived from CRF07_BC and CRF55_01B. Bayesian phylogenetic inference suggested that CRF126_0755 originated in approximately 2005-2007. The present findings reveal that the genotype composition of HIV-1 has become more complex than that of other viruses and highlight the urgent need for continuous molecular screening and epidemic surveillance within HIV-1-infected populations to advance our understanding of viral transmission mechanisms.

**Supplementary Information:**

The online version contains supplementary material available at 10.1007/s00705-024-06030-6.

Frequent HIV-1 intersubtype recombination can result in the generation of novel and complex circulating recombinant forms (CRFs) of HIV-1 and a variety of other HIV-1 variants [1]. To date, at least 32 CRFs have been reported in China, accounting for more than 80% of HIV infections [2, 3].

In recent years, the prevalence of HIV-1 among men who have sex with men (MSM) in China has been increasing. Guangdong Province has one of the highest HIV burdens in China, with more than 84,000 people living with HIV/AIDS in 2022 [4]. Molecular surveillance of HIV-1 showed that CRF07_BC, CRF01_AE, and CRF55_01B were the dominant genotypes circulating in Guangdong [5]. Recently, an increasing number of second- and third-generation recombinants have emerged among MSM in Beijing, Hebei, Henan, Sichuan, and Yunnan [2]. The cocirculation of CRF07_BC and CRF55_01B may have enabled intersubtype recombination, especially among the MSM population. In this study, we characterized a newly emerging HIV-1 CRF, CRF126_0755, which was derived from CRF07_BC and CRF55_01B, in Guangdong Province and analysed its evolutionary history.

Plasma specimens from four HIV-1-positive individuals with no epidemiological links were collected as part of pretreatment drug resistance surveillance in the cities of Guangzhou, Zhongshan, and Dongguan in Guangdong Province. The corresponding epidemiological and demographic information was downloaded from the National Information Surveillance System. Nearly full-length HIV-1 genome (NFLG) sequences from these four specimens were determined and submitted to the GenBank database under the accession numbers ON456387 to 456390 (Supplementary Table S1).

Viral RNA was extracted from plasma samples and subsequently reverse transcribed to cDNA using a SuperScript III First-Strand Synthesis System (Invitrogen, USA). Then, the two overlapping halves of the HIV-1 genome were amplified by nested polymerase chain reaction (PCR) utilizing LA Taq (TaKaRa, China), using the near-endpoint dilution method as described previously [6]. Positive products were purified and sequenced. The NFLG sequences, after assembly and trimming using the DNA sequence analysis software Sequencher V5.4.6, were 8898, 8929, 8923, and 8974 nt long, spanning from the 5' LTR to the 3' LTR, corresponding to nt 790-9469 of the HXB2 strain.

The sequences were aligned with reference sequences downloaded from the Los Alamos HIV database, using BioEdit V7.2. A maximum-likelihood (ML) phylogenetic tree was constructed using the general time-reversible (GTR) substitution model with 1000 ultrafast bootstrap replicates of to assess the reliability of the internal branches, using IQtree software V1.6.12 [7]. The ML phylogenetic tree revealed that the NFLGs formed a tight, large monophyletic cluster (bootstrap value, 100%) that was separated from known subtypes and CRFs, as shown in Fig. 1A. The sequences were analyzed using jpHMM (http://jpHMM.gobics.de/submission_hiv.html), RIP (https://www.hiv.lanl.gov/content/sequence/RIP/RIP.html), and BootScan (Simplot software V3.5.1) with a window size of 350 bp and steps of 20 bp. BootScan analysis revealed that the four NFLGs exhibited very similar recombination patterns, with the corresponding segments of the *pol*, *vif*, *vpr*, *vpu*, and *env* genes in the CRF55_01B backbone substituted by segments from CRF07_BC (Fig. 1B). The whole genome was divided into three regions with two breakpoints, corresponding to HXB2 at 2341 and 8306 (Fig. 1C). Subregion tree analysis confirmed the recombinant structure of the four NFLGs. ML phylogenetic trees indicated that segments I and III were stably clustered with the CRF07_BC lineage, with a bootstrap value of 100%. Segment I and segment III were grouped with the Chinese MSM variants in the CRF07_BC lineage (bootstrap support, 89% and 98%, respectively). Segment II was stably clustered with CRF55_01B, with a bootstrap value of 100% (Fig. 2A). The results indicated that these four NFLGs had the same recombination pattern representing a novel CRF, designated as CRF126_0755.

**Fig. 1:**
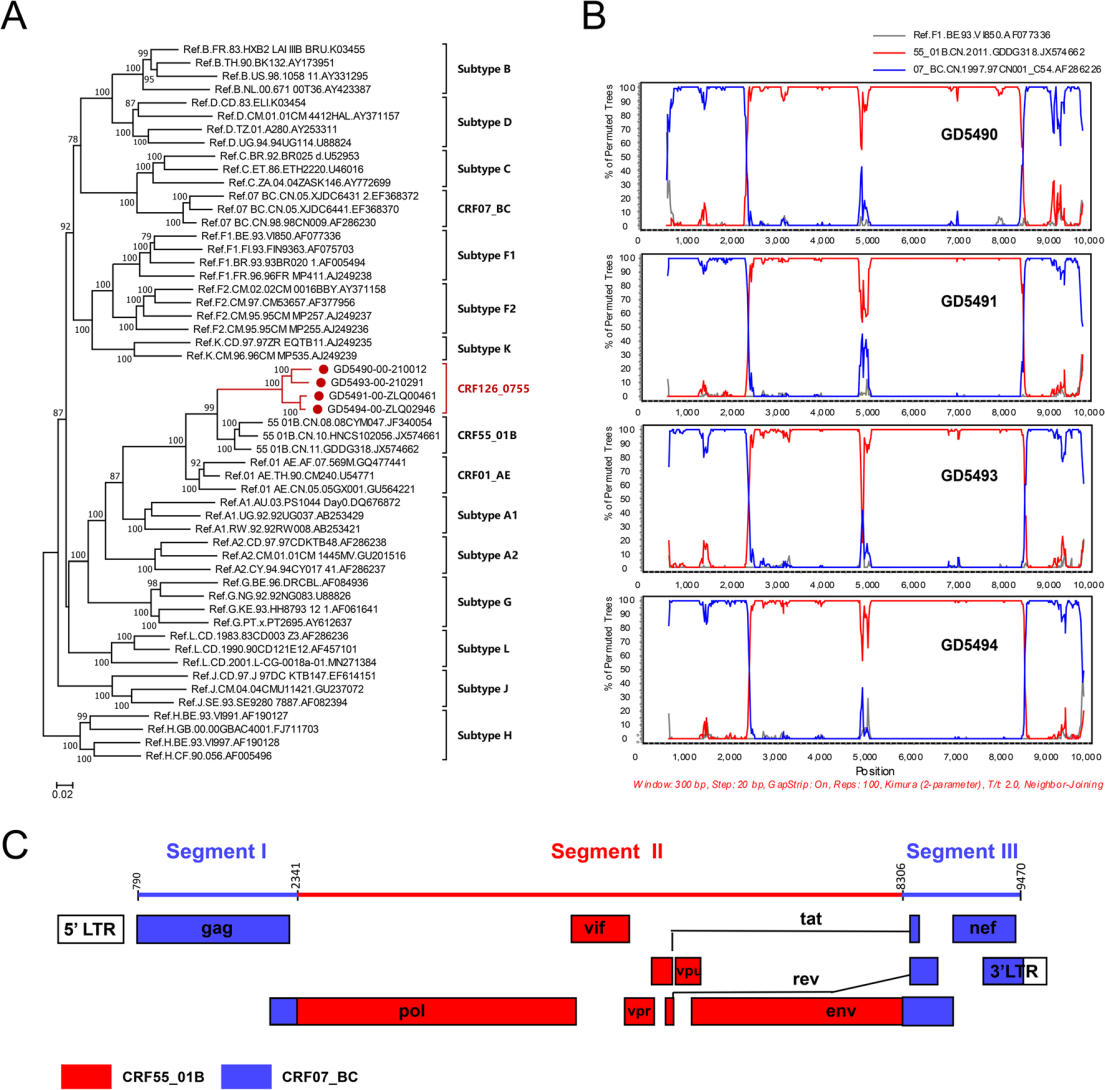
Phylogenetic and recombination analysis of CRF126_0755. (**A**) Maximum-likelihood (ML) phylogenetic tree, constructed using reference sequences downloaded from the Los Alamos National Library (LANL) HIV database (https://www.hiv.lanl.gov/), including subtypes A-D, F-H, J, K, L, CRF07_BC, and CRF55_01B, together with CRF126_0755 NFLG sequences. Each reference sequence is labeled with the HIV-1 subtype, followed by the sequence name and accession number. The scale bar represents a genetic distance of 2%. The reliability of the nodes was tested by the bootstrap method with 1000 repetitions. Branches with bootstrap values greater than 70% were considered reliable, and the values are shown at the nodes. (**B**) BootScan analysis, performed using SimPlot 3.5 software with a window size of 300 bp and step size of 20 bp. (**C**) Genome mosaic map of CRF126_0755 NFLG, generated using the LANL Recombinant HIV-1 Drawing Tool (https://www.hiv.lanl.gov/content/sequence/DRAW_CRF/recom_mapper.html)

**Fig. 2:**
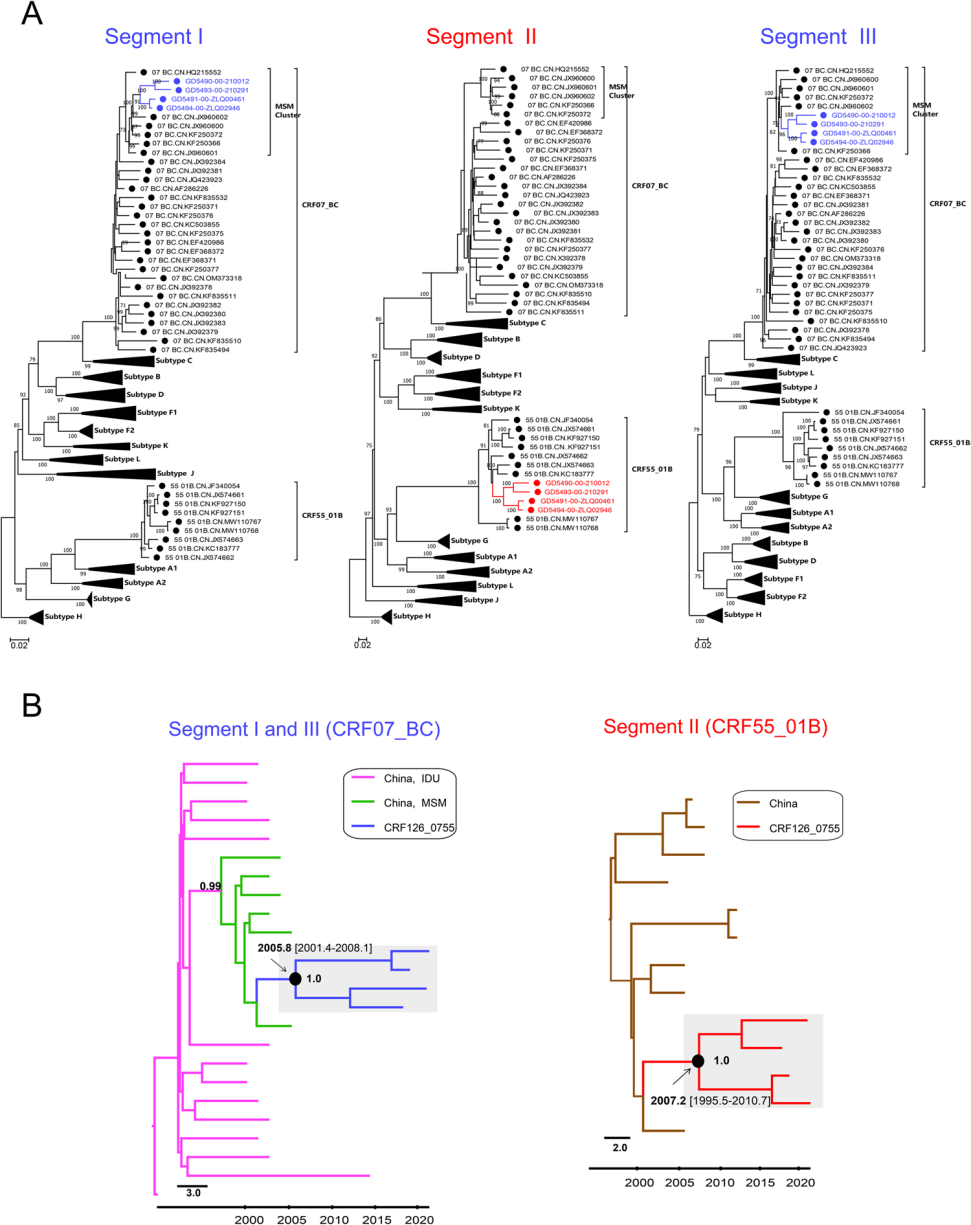
Subregion phylogenetic trees and maximum clade credibility (MCC) trees for CRF126_0755 sequence analysis. (**A**) Subregion phylogenetic trees. Maximum-likelihood (ML) phylogenetic trees of segments I, II, and III were constructed using the general time-reversible (GTR) substitution model with 1000 ultrafast bootstrap replicates, using IQtree software V1.6.12. The reference sequences were downloaded from the Los Alamos National Library (LANL) HIV database (https://www.hiv.lanl.gov/) and represent subtypes A-D, F-H, J, K, L, Chinese CRF07_BC, and Chinese CRF55_01B. The reliability of the nodes was tested by the bootstrap method with 1000 repetitions. Branches with bootstrap values greater than 70% were considered reliable, and the values are shown at the nodes. (**B**) Maximum-clade credibility (MCC) trees. Bayesian coalescent Markov chain Monte Carlo analysis was performed using BEAST 1.10.4. The MCC trees for the CRF07_BC and CRF55_01B regions of CRF126_0755 were annotated using TreeAnnotator v1.7.2 and visualized using FigTree 1.4.0. The grey area indicates CRF126_0755. The estimated time to the most recent common ancestor (tMRCA) and the posterior probability value are shown near the nodes

To investigate the origin of CRF126_0755, Bayesian coalescent Markov chain Monte Carlo (MCMC) inference [8] was performed with a chain length of 200 million and concatenated segments, using BEAST software V1.10.4. The convergence of parameters was evaluated using Tracer v1.5, and a maximum-clade-credibility (MCC) tree was constructed in TreeAnnotator v1.7.2 after discarding the first 10% of the states of each run as burn-in and visualized using FigTree v1.3.1. Bayesian coalescent analysis indicated that segments I and III of these four NFLGs clustered with Chinese MSM variants of the CRF07_BC lineage, with a posterior probability value of 0.99. The results indicated that the estimated dates of origin of the most recent common ancestors (MRCA) of CRF126_0755 were 2005.8 [95% highest probability density (HPD): 2001.4, 2008.1] and 2007.2 (95% HPD: 1995.5, 2010.7) (Fig. 2B). Therefore, HIV-1 CRF126_0755 was inferred to have originated in approximately 2005-2007.

The HIVdb program from the Stanford University HIV Drug Resistance Database (https://hivdb.stanford.edu/hivdb/bysequences/) was used to scan the sequences for drug resistance mutations (DRMs). DRMs were classified according to their associations with protease inhibitors (PIs), nucleoside reverse-transcription inhibitors (NRTIs), non-nucleoside reverse-transcription inhibitors (NNRTIs), and integrase strand transfer inhibitors (INSTIs). Drug resistance was divided into five levels, and variants with low resistance or higher were defined as having drug resistance. No PI or NRTI DRMs were identified. All four CRF126_0755 NFLGs contained the NNRTI resistance mutation V179E. In addition, one of the CRF126_0755 NFLGs (GD5493) had the INSTI accessory mutation S153A.

The amino acid sequence of the V3 loop was analysed to predict cell tropism. The online program Variable Region Characteristics (https://www.hiv.lanl.gov/content/sequence/VAR_REG_CHAR/index.html) was used to determine the length, N-linked glycosylation site, and net charge of the V3 loop. The online program Geno2pheno (https://coreceptor.geno2pheno.org) was used to predict HIV-1 co-receptors, with a false-positive rate (FPR) of 10% [9]. All viruses with FPR sequence prediction results >10% were considered R5-tropic, whereas FPRs ≤10% were considered X4-tropic. The V3 loops of all four CRF126_0755 NFLG sequences were 35 residues long, had the same net charge of +3, had an FPR between 13.0% and 46.0% (indicating R5-tropism), and had a highly conserved crown motif (GPGQ) at the tip of the V3 region, which is considered the focal point of a potent neutralizing antibody epitope (Supplementary Table S2).

CRF07_BC is the most prevalent CRF in China [3]. A new CRF07_BC cluster circulating mainly among MSM was identified as distinct from the original CRF07_BC circulating among injecting drug users (IDUs) [10]. CRF07_BC and CRF55_01B were the dominant genotypes in Guangdong Province in 2018 [5]. The CRF55_01B strain is the first CRF01_AE and B subtype recombinant strain in China [11]. Like other strains in MSM, CRF55_01B spread from Guangdong throughout the whole country, and expanded rapidly with the rapid development of the Beijing-Kowloon railways, becoming the fifth-most-common HIV-1 strain in China in approximately 2018 [11]. Recombination is an important mechanism by which novel HIV-1 variants are generated [12]. Cocirculation of these CRFs unavoidably creates additional opportunities for the generation of further complex recombinants.

Although a CRF derived from the CRF07_BC and CRF55_01B lineages has never been reported, unique recombinant forms (URFs) derived from CRF07_BC and CRF55_01B have already been identified [13–16]. These URFs have different breakpoints and differ from CRF126_0755 (Supplementary Fig. S1). The recombination patterns and genetic diversity of HIV-1 strains have become more intricate than those of other viruses, and it is important to monitor the drug resistance and pathogenicity of these highly recombinant strains. The NNRTI resistance mutation V179E, which has been detected in almost all CRF55_01B strains, was found in all four CRF126-0755 NFLGs, possibly conferring increased drug resistance [17]. A previous study of patients infected with CRF55_01B strains revealed a relatively low CD4 count and a rapid increase in plasma viral load, suggesting that is associated with a longer asymptomatic phase and a greater risk of HIV transmission [18]. Additional investigations will focus on the virological treatment and immune status of patients with this new CRF.

In summary, we have discovered a novel HIV-1 CRF, CRF126_0755, in which genes in the CRF55_01B background were replaced by CRF07_BC. This CRF was estimated to have emerged in approximately 2005-2007. The emergence of CRF126_0755 indicates that the pattern of circulating HIV-1 genotypes in Guangdong Province and southern China is more complex than previously thought. Surveillance of molecular epidemiological dynamics and drug resistance should be reinforced to limit CRF126_0755 transmission, halt disease progression, and improve prognosis.

### Supplementary Information

Below is the link to the electronic supplementary material.

Supplementary file1 (DOCX 16 KB)

Supplementary file2 (PDF 404 KB)

## Data Availability

The data that support the findings of this study are available from the corresponding author upon reasonable request.
