# Supplementary material for: Identification of a novel HIV-1 third-generation circulating recombinant form (CRF126_0755) in Guangdong, China
Source: Arch Virol. 2024 Apr 8;169(5):92. doi: 10.1007/s00705-024-06030-6 (PMC11001704; doi:10.1007/s00705-024-06030-6)
Supplement: Supplementary file 1 — Supplementary file1 (DOCX 16 KB) [file 705_2024_6030_MOESM1_ESM.docx]

**Supplementary Table1 Sociodemographic characteristics of 4 HIV-1 infected individuals**

| **Sample ID** | **Sampling data** | **Gender** | **Age** | **Residential city** | **Transmission route** | **Diagnosis date** | **Marriage status** | **Baseline CD4 T cell count (cells/mL)** | **Baseline HIV-1 RNA Viral Load (IU/mL)** | **Initial treatment regimen** | **Accession number** |
| --- | --- | --- | --- | --- | --- | --- | --- | --- | --- | --- | --- |
| GD5490-00-210012 | 2021-1-6 | male | 38 | Guangzhou | Unknown | 2020-12-28 | married | 43 | 2.82E+04 | 3TC+DTG+TDF | ON456387 |
| GD5491-00-ZLQ00461 | 2021-4-7 | male | 22 | Guangzhou | MSM | 2021-4-7 | unmarried | 549 | 1.85E+06 | 3TC+DTG+TAF | ON456388 |
| GD5493-00-210291 | 2018-12-25 | male | 40 | Dongguan | MSM | 2018-12-19 | unmarried | 374 | 7.23E+04 | 3TC+EFV+TDF | ON456389 |
| GD5494-00-ZLQ02946 | 2019-7-25 | male | 23 | Zhongshan | HET | 2019-6-6 | unmarried | 452 | 2.60E+04 | 3TC+EFV+TDF | ON456390 |

**Supplementary Table2 Cell tropism prediction based on the amino acid sequence of the CRF126_0755 V3 loop**

| **Sample ID** | **Length** | **NX[ST]^1^** | **Charge^2^** | **Sequence^3^** | **FPR^4^** |
| --- | --- | --- | --- | --- | --- |
| GD5490-00-210012 | 35 | 1 | 3 | CTRPANNTRTGITIGPGQTWYRTGDIIGDIRKAFC | 28.8% |
| GD5491-00-ZLQ00461 | 35 | 1 | 3 | CSRPANNTRTGVIIGPGQTWYRTGDIIGDIRKAFC | 46.0% |
| GD5493-00-210291 | 35 | 1 | 3 | CTRTANNTRTGITIGPGQTWYRTGEIIGDIRRAFC | 13.0% |
| GD5494-00-ZLQ02946 | 35 | 1 | 3 | CTRPANNTRTGVIIGPGQTWYRTGDIIGDIRKAFC | 37.2% |

NOTE：

^1^NX[ST]: the sequence pattern of N-linked glycosylation sites, where X can be any amino acid except P

^2^Charge: net charge = (R + K)–(D + E), KR = +, DE = -

^3^Residues with a grey background showed the crown motif (amino acids 15–18) in the tip of V3 region, which was considered as the focal point of the potent neutralising antibody epitope

^4^FPR: false positive rate, the probability of classifying an R5-virus falsely as X4
