# Supplementary figures and images for: Identification of a novel HIV-1 third-generation circulating recombinant form (CRF126_0755) in Guangdong, China
Source: Arch Virol. 2024 Apr 8;169(5):92. doi: 10.1007/s00705-024-06030-6 (PMC11001704; doi:10.1007/s00705-024-06030-6)

CRF126\_0755

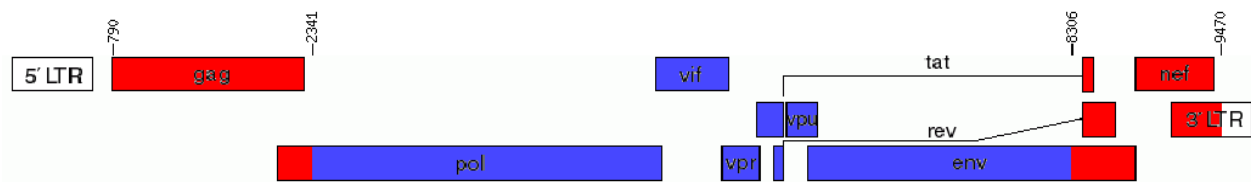

SZ44LS7251

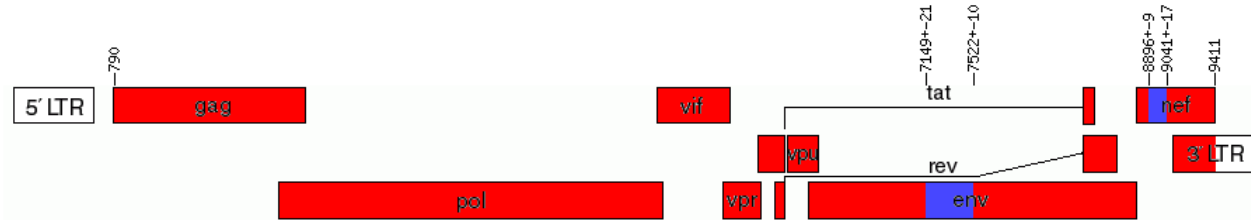

ZJCIQ15005

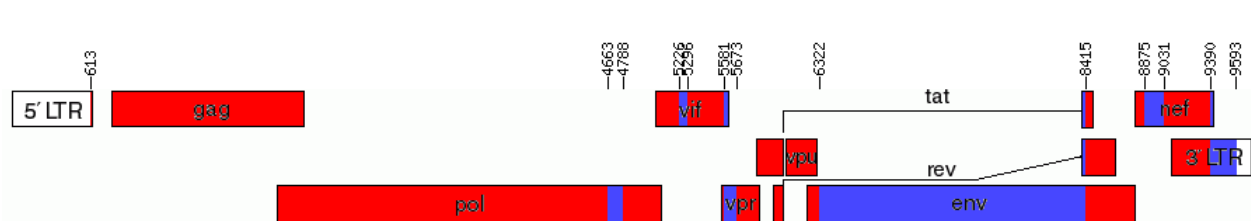

S15

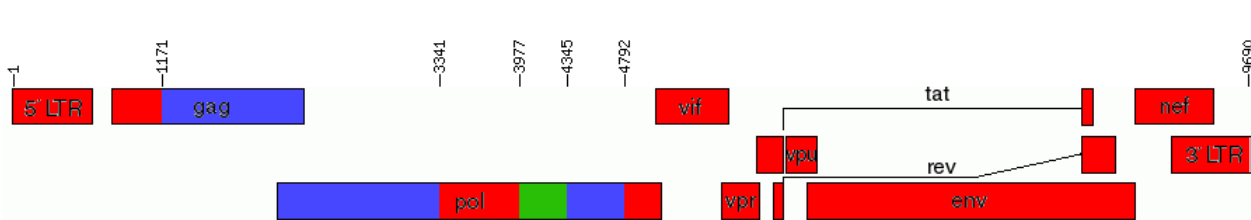

JM05

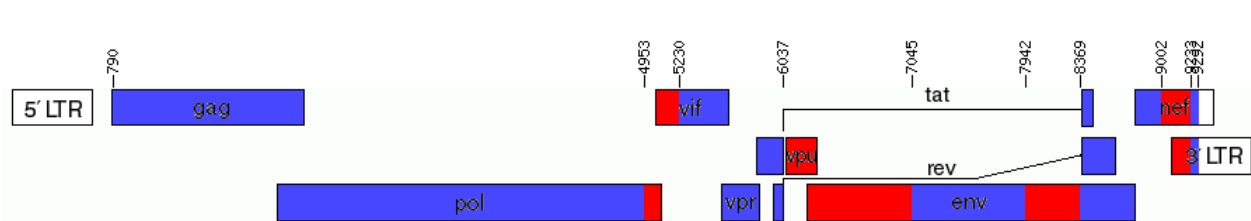

Supplement: Supplementary file 2 — Supplementary file2 (PDF 404 KB) [file 705_2024_6030_MOESM2_ESM.pdf]
